# Post return of spontaneous circulation factors associated with mortality in pediatric in-hospital cardiac arrest: a prospective multicenter multinational observational study

**DOI:** 10.1186/s13054-014-0607-9

**Published:** 2014-11-03

**Authors:** Jesús López-Herce, Jimena del Castillo, Martha Matamoros, Sonia Canadas, Ana Rodriguez-Calvo, Corrado Cecchetti, Antonio Rodríguez-Núnez, Ángel Carrillo, Iberoamerican Pediatric Cardiac Arrest Study Network RIBEPCI

**Affiliations:** Pediatric Intensive Care Department, Hospital General Universitario Gregorio Maranón, Dr Castelo 47, 28009 Madrid, Spain; Instituto de Investigación Sanitaria del Hospital Gregorio Marañón de Madrid, Red de Salud Materno Infantil y del Desarrollo (Red SAMID), Dr Castelo 47, 28009 Madrid, Spain; Hospital Escuela, Boulevard Suyapa, Tegucigalpa, Honduras; Hospital Valle de Hebrón, Passeig Vall d’Hebron, 119-129 08035 Barcelona, Spain; Hospital Nino Jesús, Hungría 750, 4000 San Miguel de Tucumán, Argentina; Ospedale Bambinu Gesu, Via della Torre di Palidoro, 00050 Fiumicino Roma, Italy; Hospital Clínico Universitario de Santiago de Compostela, Travesía de Choupana, s/n, 15706 A Coruña, Spain; Iberoamerican Pediatric Cardiac Arrest Study Network RIBEPCI, Dr Castelo 47, 28009 Madrid, Spain

## Abstract

**Introduction:**

Most studies have analyzed pre-arrest and resuscitation factors associated with mortality after cardiac arrest (CA) in children, but many patients that reach return of spontaneous circulation die within the next days or weeks. The objective of our study was to analyze post-return of spontaneous circulation factors associated with in-hospital mortality after cardiac arrest in children.

**Methods:**

A prospective multicenter, multinational, observational study in 48 hospitals from 12 countries was performed. A total of 502 children aged between 1 month and 18 years with in-hospital cardiac arrest were analyzed. The primary endpoint was survival to hospital discharge. Univariate and multivariate logistic regression analyses were performed to assess the influence of each post-return of spontaneous circulation factor on mortality.

**Results:**

Return of spontaneous circulation was achieved in 69.5% of patients; 39.2% survived to hospital discharge and 88.9% of survivors had good neurological outcome. In the univariate analysis, post- return of spontaneous circulation factors related with mortality were pH, base deficit, lactic acid, bicarbonate, FiO2, need for inotropic support, inotropic index, dose of dopamine and dobutamine at 1 hour and at 24 hours after return of spontaneous circulation as well as Pediatric Intensive Care Unit and total hospital length of stay. In the multivariate analysis factors associated with mortality at 1 hour after return of spontaneous circulation were PaCO_2_ < 30 mmHg and >50 mmHg, inotropic index >14 and lactic acid >5 mmol/L. Factors associated with mortality at 24 hours after return of spontaneous circulation were PaCO_2_ > 50 mmHg, inotropic index >14 and FiO_2_ ≥ 0.80.

**Conclusions:**

Secondary in-hospital mortality among the initial survivors of CA is high. Hypoventilation, hyperventilation, FiO_2_ ≥ 0.80, the need for high doses of inotropic support, and high levels of lactic acid were the most important post-return of spontaneous circulation factors associated with in-hospital mortality in children in our population.

**Electronic supplementary material:**

The online version of this article (doi:10.1186/s13054-014-0607-9) contains supplementary material, which is available to authorized users.

## Introduction

Most studies have analyzed pre-arrest and resuscitation factors associated with mortality after cardiac arrest (CA) in children [1-13]. Previous studies have shown that lower human development index of countries, characteristics of the hospital, CA that occurred out of hospital and out of the Pediatric Intensive Care Unit (PICU), oncohematologic disease, treatment with inotropic drugs at the time of the CA, CA due to neurological disease or sepsis, time to the initiation of resuscitation, asystole as the initial electrocardiographic (ECG) rhythm, need for adrenaline, bicarbonate or fluid expansion and the duration of cardiopulmonary resuscitation (CPR) are associated with higher mortality [1-13].

An important percentage of patients that reach return of spontaneous circulation (ROSC) die within the next days or weeks. However, there are no multicenter, multinational prospective studies on CA in children to have analyzed post-ROSC prognostic factors. In previous studies performed on the same prospective register we have analyzed the pre-arrest and resuscitation factors [11] and the ventilation and oxygenation factors associated with mortality [14]. The objective of the present study was to analyze the factors affecting mortality and neurological outcome of in-hospital CA in children. The hypothesis was that respiratory status and hemodynamic status are the most important prognosis factors after ROSC in children.

## Methods

An open multicenter prospective study was designed and information and an invitation to participate were sent to the pediatric departments and PICUs of hospitals in Latin-American countries, Spain, Portugal, and Italy. The study was approved by local Institutional Review Boards (Additional file 1). Registration on the website [15] was necessary to participate in the study. Consent of parents of patients was not considered necessary because it was an observational study during and after CA and it is necessary to obtain data immediately.

A protocol was drawn up in accordance with the Utstein style [16,17]. Children aged from 1 month to 18 years who suffered in-hospital CA between December 2007 and December 2009 were included. CA was defined by the presence of all the following signs: unresponsiveness, apnea, absence of signs of life and absence of a palpable central pulse or bradycardia with less than 60 beats per minute (bpm) with poor perfusion in infants requiring external cardiac compressions and assisted ventilation.

All data were entered via a secure, encrypted website and were electronically submitted to the coordinating center. That center performed a review of all records to ensure data quality, and site investigators were queried to complete missing data and resolve discrepancies.

Patient-related variables and arrest and life support-related parameters have been previously published [11] and also the relationship between ventilation and oxygenation with mortality [14]. In the present study we analyzed the influence on survival of several post-ROSC parameters, such as arterial gasometry and lactic acid at the first hour and 24 hours after ROSC, the need for mechanical ventilation, recovery of spontaneous breathing (although mechanical ventilation could be needed), the need for vasoactive drugs and doses of vasoactive drugs, vasoactive-inotropic index (VIS) [18], and ECG rhythm after ROSC. Hospital course and clinical and neurological status at hospital discharge according to the pediatric cerebral performance category [PCPC] were registered [19]. Variable definitions were based on Utstein-style guidelines [16,17]. The primary endpoint was survival to hospital discharge. The secondary outcome measure was neurological status at hospital discharge; a good neurological status was defined as a PCPC score of 1 or 2 [19].

Statistical analyses were conducted using SPSS software version 18.1 (SPSS Inc, Chicago, IL, USA). Outcomes were compared between groups using the chi-square (*χ*^2^) test or Fisher’s exact test for categorical variables. Univariate and multivariate logistic regression analysis was performed to assess the influence of each one of the factors on mortality. A logistic regression model was constructed for variables at 1 hour and at 24 hours after ROSC. All individual factors with statistical significance in the univariate analysis and *P* <0.1 were eligible for inclusion in the logistic regression model. Receiver operator characteristic (ROC) curves were used to decided cutoffs values for VIS and lactic acid. Ventilation and oxygenation cutoff values were chosen according to previous studies [20,21] and normal limits of pH, ventilation and oxygenation. Finally a logistic regression model was constructed including patient-related variables, arrest- and life support-related parameters, and post-ROSC parameters. Adjusted odds ratios (OR) and 95% confidence intervals (CI) were calculated for each model. ROC curves were used to assess the predictive capacity of each model.

## Results

Forty-eight hospitals from twelve countries participated in the study. The analysis included 563 episodes of in-hospital CA in 502 patients. CA occurred in the PICU in 50% of cases, in the emergency department in 26.8%, and in other hospital areas in 23.2%.

Return of spontaneous circulation (ROSC) for more than 20 minutes was achieved in 349 patients (69.5%), but 152 (30.3%) patients died later in hospital due to new CA (32.9%), multiple organ dysfunction (27%), limitation of medical therapy (25%) or brain death (15.1%): 197 patients (39.2%) survived to hospital discharge. Five patients were rescued with extracorporeal membrane oxygenation (ECMO) during CPR and four of them survived to hospital discharge. The characteristics of the 502 patients, pre-arrest factors, and cardiac arrest and resuscitation factors associated with mortality have been previously published [11].

### Post-ROSC factors associated with mortality

Table 1 shows the comparison between survivors and non-survivors in post-ROSC factors. Non-survivors had lower pH, higher base excess, higher lactic acid levels and higher inspired oxygen fraction (FiO_2_) at 1 and 24 hours after ROSC than survivors. A greater percentage of non-survivors needed inotropic support, and they required higher doses of dopamine and dobutamine, and had a higher inotropic score than survivors did. Nevertheless, the percentage of patients receiving milrinone was lower in non-survivors than in survivors. When patients without inotropic support before CA were analyzed separately, children who required inotropic support after ROSC had 42.1% higher mortality than those who did not need it 26.9% (*P* =0.036). Finally, the length of PICU stay and total hospital stay was shorter in non-survivors.

**Table 1:** **Comparison between survivors and non-survivors**

	**Number of patients**	**Non-survivors**	**Survivors**	***P*** **-value**
		**Median (IQR)**	**Median (IQR)**	
**Gasometry at 1 h**				
pH	259	7.23 (7.03 to 7.35)	7.29 (7.17 to 7.38)	**0.004**
PaO_2_, mmHg	253	78.5 (45.0 to 125.0)	81.0 (47.0 to 136.0)	0.476
PaO_2_/FiO_2_	231	85.5 (50.75 to 189.5)	111 (63 to 242)	0.101
PaCO_2_, mmHg	255	46 (32 to 63)	42 (34 to 52)	0.314
HCO_3_ mEq/L	241	18 (12.0 to 24.5)	21 (15 to 25)	0.064
BE	233	-8 (-2 to -15)	-6 (-0.75 to -12)	**0.049**
Lactic acid, mmol/L	136	7.2 (7 to 13)	4.1 (1.89 to 8)	**0.002**
FiO_2_	249	100 (72.5 to 100)	100 (50 to 100)	**0.034**
**Gasometry at 24 h**				
pH	209	7.35 (7.26 to 7.42)	7.40 (7.34 to 7.45)	**0.001**
PaO_2_, mmHg	205	77 (50 to 124)	82 (49.75 to 119.25)	0.924
PaO_2_/FiO_2_	178	125 (70.25 to 242.08)	111 (63 to 242)	**0.031**
PaCO_2_, mmHg	208	42.5 (34.25 to 53.5)	41 (35 to 46)	0.175
HCO_3_, mEq/L	205	22 (19 to 27)	25 (21 to 29)	**0.009**
BE	186	-2 (-5 to 3.1)	1 (-3 to 5)	**0.031**
Lactic acid, mmol/L	123	2 (1.2 to 6.47)	1.4 (0.9 to 2.25)	**0.007**
FiO_2_	191	80(50 to 100)	45 (30 to 65)	**0.001**
Lactic acid clearance, %	99	60 (25 to 75)	68.63 (27.81 to 84.70)	**0.385**
**Mechanical ventilation after ROSC, %**	215	94.4	93.5	0.756
**Vasoactive treatment**				
Patients with vasopressors after ROSC, %	173	76.3	63.9	**0.039**
Inotropic score after ROSC	154	80.7 (121.6)	38.3 (78.7)	**0.001**
**Length of stay**				
Days in the PICU	224	7.9 (14.7)	17.4 (19.4)	**0.001**
Days in hospital ward	118	4.5 (6.9)	17.3 (19.4)	**0.001**

Significant values marked in bold. BE, base excess; PICU, Pediatric ICU; ROSC, return of spontaneous circulation; FiO_2,_ inspired oxygen fraction; PaO_2,_ arterial partial pressure of oxygen.

Table 2 summarizes post-ROSC factors and their relationship with survival to hospital discharge in the univariate regression analysis. The need for inotropic support, an inotropic index greater than 14, the absence of spontaneous breathing, PaCO_2_ < 30 mmHg or >50 mmHg, FiO_2_ ≥ 0.80 and lactic acid levels >5 mmol/L at 1 hour after ROSC, as well as pH <7.30, PaCO_2_ > 50 mmHg and FiO_2_ > 50% at 24 hours after ROSC were significantly associated with higher mortality rates. There were no significant differences in post-ROSC PaO_2_ between survivors and non-survivors patients, even when the 24 patients with cyanotic heart disease and 6 patients on ECMO were excluded of analysis.

**Table 2:** **Univariate analysis of mortality according to post-return of spontaneous circulation factors**

	**Patients, number**	**Patients, %**	**Mortality, %**	**Odds ratio**	**95% CI**	***P*** **-value**
**Mechanical ventilation**						
No	42	6.1	41.2	1		
Yes	173	93.9	45	3.273	0.713 to 15.027	0.127
**Vasoactive drugs**						
No	79	31.3	29.1	1		
Yes	173	68.7	42.8	1.820	1.028 to 3.222	**0.040**
**Vasoactive-inotropic score**						
<14	62	40.3	29	0.390		
>14	92	59.7	51	2.564	1.288 to 5.050	**0.007**
**Electrocardiographic rhythm after ROSC**						
Sinus rhythm	230	72.8	43.5	1		
Other rhythms	86	27.2	53.5	1.495	0.909 to 2.459	0.113
**Recovery of spontaneous breathing**						
Yes	86	26	22.1	1		
No	245	74	57.1	4.694	2.659 to 8.333	**0.001**
**pH 1 h**						
7.30 to 7.50	96	37.1	37.5	1		
<7.30	154	59.5	48.7	1.582	0.941 to 2.662	0.084
>7.50	9	3.5	22.2	0.476	0.094 to 2.418	0.371
**PaO**_**2**_ **1 h**						
60 to 200 mmHg	123	48.6	44.7	1		
<60 mmHg	96	37.9	43.8	0.962	0.562 to 1.646	0.887
**>**200 mmHg	34	13.4	32.4	0.591	0.265 to 1.318	0.199
**PaCO**_**2**_ **1 h**						
30 to 50 mmHg	133	52.2	33.1	1		
<30 mmHg	37	14.5	62.2	3.323	1.560 to 7.079	**0.002**
>50 mmHg	85	33.3	52.9	2.276	1.302 to 3.978	**0.004**
**CO**_**3**_ **H 1 h**						
20 to 26 mEq/L	75	31.1	36	1		
<20 mEq/L	118	49	46.6	1.552	0.857 to 2.812	0.147
>26 mEq/L	48	19.9	41.7	1.27	0.604 to 2.669	0.528
**Base excess 1 h**						
+4 to -4	48	20.6	37.5	1		
<-4	154	66.1	46.8	1.463	0.753 to 2.844	0.261
> + 4	31	13.3	41.9	1.204	0.479 to 3.027	0.694
**FiO**_**2**_ **1 h**						
<0.50	42	16.7	12.1	1		
0.50 to 0.79	41	16.3	12.9	1.150	0.468 to 2.847	0.756
≥0.80	169	67.1	75	2.120	1.044 to 4.311	**0.038**
**PaO**_**2**_ **/FiO**_**2**_ **1 h**						
>300	166	16.7	45.5	1		
200 to 300	37	11.7	25.8	0.417	0.154 to 1.134	0.087
<200	28	71.6	46	1.024	0.530 to 1.978	0.945
**Lactic acid 1 h**						
<2 mmol/L	27	19.9	22.2	1		
2 to 5 mmol/L	39	28.7	28.2	1.375	0.438 to 4.318	0.585
>5 mmol/L	70	51.5	51.4	3.706	1.335 to 10.29	**0.012**
**pH 24 h**						
7.30 to 7.50	158	75.6	27.8	1		
<7.30	39	18.7	53.8	3.023	1.472 to 6.205	**0.003**
>7.50	70	5.7	33.3	1.295	0.371 to 4.520	0.685
**PaO**_**2**_ **24 h**						
60 to 200 mmHg	121	59	32.2	1		
<60 mmHg	70	34.1	28.6	0.841	0.442 to 1.601	0.598
**>**200 mmHg	14	6.8	57.1	2.803	0.910 to 8.636	0.073
**PaCO**_**2**_ **24 h**						
30 to 50 mmHg	123	68.3	25.2	1		
<30 mmHg	14	7.8	35.7	1.649	0.513 to 5.294	0.401
>50 mmHg	43	23.9	51.2	3.109	1.508 to 6.409	**0.002**
**CO**_**3**_ **H 24 h**						
20 to 26 mEq/L	90	43.9	36.7	1		
<20 mEq/L	41	20	43.9	1.352	0.638 to 2,865	0.432
>26 mEq/L	74	36.1	24.3	0.555	0.281 to 1,099	0.091
**Base excess 24 h**						
+4 to -4	90	48.4	30	1		
<-4	52	28	40.4	1.581	0.774 to 3.229	0.209
> + 4	44	23.7	22.7	0.686	0.297 to 1.585	0.378
**FiO**_**2**_ **24 h**						
<0.50	80	41.9	22.1			
0.50 to 0.79	48	25.1	25	2.376	1.051 to 5.371	**0.038**
≥0.80	63	33	52.9	5.778	2.726 to 12.245	**0.001**
**PaO**_**2**_ **/FiO**_**2**_ **24 h**						
>300	116	18.2	21.6	1		
200 to 300	34	16.3	30.3	2.327	0.988 to 5.481	0.053
< 00	28	65.5	39.1	1.576	0.536 to 4.636	0.408
**Lactic acid 24 h**						
<2 mmol/L	73	59.3	23.3	1		
2 to 5 mmol/L	29	23.6	37.9	2.013	0.798 to 5.081	0.139
>5 mmol/L	21	17.1	47.6	2.995	1.086 to 8.254	**0.034**
**Lactic acid clearance**						
>50%	56	55.9	28.8	1		
≤50%	43	44.1	30.8	1.099	0.497 to 2.433	0.841

Significant values marked in bold. Lactid acid clearance: (lactate after ROSC minus lactate 24 hours after ROSC) × 100/lactate after ROSC. ROSC, return of spontaneous of circulation; FiO_2,_ inspired oxygen fraction; PaO_2,_ arterial partial pressure of oxygen.

In the multivariate analysis, factors associated with mortality at 1 hour after ROSC were PaCO_2_ < 30 mmHg and >50 mmHg, inotropic index >14 and lactic acid >5 mmol/L (Table 3). Factors associated with mortality at 24 hours after ROSC were PaCO_2_ > 50 mmHg, inotropic index >14 and FiO2 ≥ 0.8 (Table 3).

**Table 3:** **Multivariate logistic regression study including mortality risk factors at 1 hour and 24 hours after return of spontaneous circulation**

	**Odds ratio**	**95% CI**	***P*** **-value**
**1 hour after return of spontaneous circulation**			
PaCO_2_ < 30 mmHg	2.640	1.190 to 5.857	0.017
PaCO_2_ > 50 mmHg	1.950	1.063 to 3.576	0.031
Lactic acid >5 mmol/L	2.021	0.926 to 4.413	0.077
Vasoactive-inotropic score >14	2.454	1.252 to 4.810	0.009
**24 hours after ROSC**			
PaCO_2_ > 50 mmHg	2.541	1.156 to 5.587	0.020
FiO_2_ ≥ 0.80	3.864	1.698 to 8.794	0.001
Vasoactive-inotropic score >14	2.070	1.008 to 4.249	0.047

**Figure 1:**
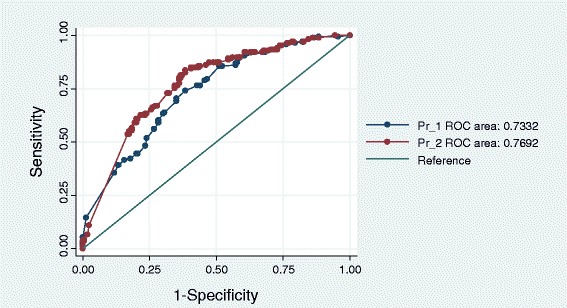
**Receiver operator characteristic (ROC) curves of mortality-associated factors at 1 hour after return of spontaneous circulation (ROSC) (area under the curve (AUC) 0.733, CI (0.681 to 0.785);** ***P*** **=0.001) and at 24 hours after ROSC (AUC 0.769, CI (0.720 to 0.819);** ***P*** **=0.001).**

The logistic regression model at 1 hour after ROSC had an AUC of 0.733 (CI 0.681 to 0.785; *P* =0.001). The logistic regression model at 24 hours after ROSC had an AUC of 0.769 (CI 0.720 to 0.819; *P* =0.001), (Figure 1).

### Post-ROSC factors associated with neurological outcome

Neurological status at hospital discharge was assessed in 120 patients (60.9%), and 107 of them (89%) had a normal neurological status or showed mild disability (PCPC l or 2). PCPC before CA and at hospital discharge was compared. Only 2.8% of patients with PCPC 1 or 2 before CA presented a PCPC >2 at hospital discharge.

When comparing patients with good and bad neurological outcome (PCPC >2), at 1 hour after ROSC those with a bad outcome had significantly lower levels of bicarbonate (19.1 (7.4) mEq/L versus 21.8 (6.9) mEq/L; *P* =0.025), higher lactic acid levels (8.1 (6.5) mmol/L versus 5.8 (11.3) mmol/L; *P* =0.003) and higher base excess (BE) (-7.1 (9.4) mEq/L versus -3.4 (9.6) mEq/L; *P* =0.042) (Table 4). A higher percentage of patients with bad neurological outcome received dobutamine (40.9%) than those with good neurological outcome (19%) *P* =0.007 (Table 4).

**Table 4:** **Comparison between patients with pediatric cerebral performance category (PCPC) 1 to 2 and those with PCPC >2**

	**PCPC 1 to 2**	**PCPC >2**	***P*** **-value**
	**Mean (SD)**	**Mean (SD)**	
**Gasometry 1 h**			
pH	7.29 (0.14)	7.24 (0.17)	0.120
PaO_2_, mmHg	118.0 (104.9)	88.3 (58.8)	0.330
PaO_2_/FiO_2_	175.1 (143.1)	121.2 (89.8)	0.111
PaCO_2_, mmHg	46.1 (14.5)	45.6 (19.4)	0.297
CO_3_H mEq/L	21.8 (6.9)	19.1 (7.4)	**0.025**
Base excess	-3.4 (9.6)	-7.1 (9.4)	**0.042**
Lactic acid, mmol/L	5.8 (11.3)	8.1 (6.5)	**0.003**
FiO_2_	77.9 (27.8)	76.8 (26.8)	0.867
**Gasometry 24 h**			
pH	7.39 (0.08)	7.39 (0.12)	0.546
PaO_2_, mmHg	96.7 (66.0)	77.4 (40.3)	0.094
PaO_2_/FiO_2_	207.1 (132.7)	162.5 (119.8)	0.053
PaCO_2_, mmHg	42.7 (10.7)	43.8 (13.1)	0.681
CO_3_H, mEq/L	25.2 (5.0)	26.2 (6.7)	0.389
Base excess	0.6 (5.2)	1.5 (7.7)	0.710
Lactic acid, mmol/L	2.2 (2.9)	6.8 (20.7)	0.405
FiO_2_	53.3 (24.4)	57.9 (26.4)	0.425
Lactic acid clearance, %	37.1 (81.9)	72.6 (19.2)	**0.009**
**Mechanical ventilation, %**	94.4	97.4	0.667
**Vasoactive treatment**			
Patients with pressors after ROSC, %	57.9	77.5	**0.033**
Inotropic score after ROSC	45.3 (78.4)	36.4 (52.6)	0.693
Patients with dopamine, %	34	43.2	0.349
Dopamine dose, mcg/kg/min	9.4 (4.9)	10.7 (5.9)	0.518
Patients with dobutamine, %	19	40.9	**0.007**
Dobutamine dose, mcg/kg/min	13.7 (8.1)	9.2 (4.9)	0.070
Patients with adrenaline, %	20	18.2	1.000
Adrenaline dose, mcg/kg/min	0.6 (0.6)	0.4 (0.1)	0.667
Patients with noradrenaline, %	12	13.6	0.789
Noradrenaline dose, mcg/kg/min	0.8 (0.7)	1.3 (0.9)	0.312
Patients with milrinone, %	20	11.4	0.241
Milrinone dose, mcg/kg/min	0.8 (0.2)	0.8 (0.3)	0.812
**Length of stay**			
Days in the Pediatric ICU	17.3 (17.9)	22.3 (24.6)	0.610
Days in hospital ward	16.3 (18.1)	21.0 (26.0)	0.648

Significant values marked in bold. ROSC, return of spontaneous circulation.

The univariate analysis showed that dobutamine administration and lactic acid levels >5 mmol/L at 1 hour, and pH >7.50, PaCO_2_ > 50 mmHg and BE >4 mEq/L at 24 hours after ROSC were associated with poor neurological evolution (Table 5).

**Table 5:** **Univariate analysis of bad neurologic evolution (pediatric cerebral performance category (PCPC) >2) according to post-return of spontaneous circulation (ROSC) factors**

	**Patients, %**	**PCPC >2, %**	**Odds ratio**	**95% CI**	***P*** **-value**
**Patients**					
**Mechanical ventilation**					
No	4.7	16.7	1.000		
Yes	95.3	30.9	2.235	0.252 to 19.791	0.470
**Vasoactive drugs**					
No	42.1	18.4	1.000		
Yes	57.9	36	1.739	0.793 to 3.186	0.167
**Adrenaline**					
No	80.6	31	1.000		
Yes	19.4	28.6	0.889	0.358 to 2.207	0.800
**Noradrenaline**					
No	87.5	30.2	1.000		
Yes	12.5	33.3	1.158	0.405 to 3.313	0.785
**Dopamine**					
No	63.2	27.5	1.000		
Yes	36.8	35.8	1.475	0.714 to 3.049	0.294
**Dobutamine**					
No	74.3	24.3	1.000		
Yes	25.7	48.6	2.951	1.351 to 6.448	**0.007**
**Milrinone**					
No	82.6	32.8	1.000		
Yes	17.4	20	0.513	0.179 to 1.469	0.213
**Vasoactive-inotropic score**					
<14	48.1	43.2	1.000		
>14	51.9	35	0.707	0.282 to 1.772	0.459
**Echocardiographic rhythm after ROSC**					
Sinus rhythm	74	27.8	1.000		
Other rhythms	26	36.8	1.497	0.685 to 3.274	0.312
**Recovery of spontaneous breathing**					
Yes	65.3	25.5	1.000		
No	34.7	33.3	1.340	0.648 to 2.767	0.430
**pH 1 h**					
7.30 to 7.50	40.8	21.6	1.000		
<7.30	54.4	32.4	1.739	0.752 to 4.023	0.196
>7.50	4.8	33.3	1.818	0.293 to 11.265	0.521
**PaO**_**2**_ **1 h**					
60 to 200 mmHg	45.2	33.3	1.000		
<60 mmHg	40.5	29.4	0.833	0.368 to 1.885	0.661
**>**200 mmHg	14.3	16.7	0.400	0.103 to 1.553	0.186
**PaCO**_**2**_ **1 h**					
30 to 50 mmHg	62.1	23.4	1.000		
<30 mmHg	9.7	50	3.278	0.940 to 11.425	0.062
>50 mmHg	28.2	37.1	1.937	0.815 to 4.601	0.134
**CO**_**3**_ **H 1 h**					
20 to 26 mEq/L	34.7	23.8	1.000		
<20 mEq/L	44.6	38.9	2.036	0.831 to 4.991	0.120
>26 mEq/L	20.7	20	0.800	0.239 to 2.683	0.718
**BE**					
+4 to -4	24.8	25	1.000		
<-4	61.1	33.3	1.500	0.557 to 4.041	0.423
> + 4	14.2	18.8	0.692	0.152 to 3.163	0.635
**FiO**_**2**_ **1 h**					
<0.50	50.9	25.5	1.000		
0.50 to 0.79	25.9	32.1	1.125	0.324 to 3.909	0.853
≥0.80	23.1	44	1.125	0.409 to 3.097	0.820
**PaO**_**2**_ **/FiO**_**2**_ **1 h**					
>300	15.9	16.7	1.000		
200 to 300	12.4	14.3	3.049	0.083 to 11.149	0.092
<200	71.7	34.6	1.286	0.224 to 7.370	0.778
**Lactic acid 1 h**					
<2 mmol/L	21.3	6.2	1.000		
2 to 5 mmol/L	37.3	25	3.520	0.675 to 18.366	0.135
>5 mmol/L	41.3	41.9	9.263	1.883 to 45.560	**0.006**
**pH 24 h**					
7.30 to 7.50	80.6	26	1.000		
<7.30	12.9	43.8	2.214	0.749 to 6.545	0.151
>7.50	6.5	62.5	4.744	1.059 to 21.248	**0.042**
**PaO**_**2**_ **24 h**					
60 to 200 mmHg	58.5	29.2	1.000		
< 60 mmHg	36.6	37.8	1.474	0.670 to 3.243	0.334
**>**200 mmHg	4.9	0	0		
**PaCO**_**2**_ **24 h**					
30 to 50 mmHg	76.9	24.1	1.000		
<30 mmHg	5.6	50	3.150	0.589 to 16.859	0.180
>50 mmHg	17.6	52.6	3.500	1.248 to 9.819	**0.017**
**CO**_**3**_ **H 24 h**					
20 to 26 mEq/L	43	26.9	1.000		
<20 mEq/L	15.7	26.3	0.969	0..295 to 3.189	0.959
>26 mEq/L	41.3	36	1.527	0.658 to 3.544	0.325
**BE 24 h**					
+4 to -4	50	19.6	1.000		
<-4	23.2	38.5	2.557	0.914 to 7.155	0.774
> + 4	26.8	46.7	3.580	1.351 to 9.482	**0.010**
**FiO**_**2**_ **24 h**					
<0.50	21.4	28	1.000		
0.50 to 0.79	19.7	30.4	1.387	0.511 to 3.765	0.521
≥0.80	59	30.4	2.301	0.850 to 6.229	**0.100**
**PaO**_**2**_ **/FiO**_**2**_ **24 h**					
>300	21	13.6	1.000		
200 to 300	17.1	22.2	3.128	0.974 to 10.049	0.056
<200	61.9	40	1.312	0.284 to 6.067	0.728
**Lactic acid 24 h**					
<2 mmol/L	66.7	26.9	1.000		
2 to 5 mmol/L	20.5	31.2	1.234	0.364 to 4.187	0.736
>5 mmol/L	12.8	40	1.810	0.444 to 7.380	0.408

Significant values marked in bold.

In the multivariate analysis the only factor associated with poor neurological outcome at 1 hour after ROSC was lactic acid above 5 mmol/L (OR 9.902, CI (1.992 to 51.008); *P* =0.006). None of the factors at 24 hours after ROSC showed statistical significance in the multivariate analysis.

### Pre-arrest, resuscitation and post-ROSC factors

The multivariate analysis including pre-arrest factors, resuscitation factors and post-ROSC factors is shown in Table 6. Factors associated with in-hospital mortality were hemato-oncologic illness, neurologic cause of arrest, CA in the emergency department, treatment with inotropic drugs before CA, administration of sodium bicarbonate, PaCO_2_ < 30 mmHg 1 hour after ROSC, PaCO_2_ > 50 mmHg one hour after ROSC and FiO_2_ ≥ 0.80 24 hours after ROSC.

**Table 6:** **Multivariate logistic regression study including pre-arrest, resuscitation and post-return of spontaneous circulation (ROSC) mortality risk factors**

**Mortality risk factors**	**Odds ratio**	**95% CI**	***P*** **-value**
Hemato-oncologic illness	2.633	1.072 to 6.469	0.035
Neurologic cause of cardiac arrest	5.528	1.726 to 17.701	0.004
Place of arrest (emergency department)	3.170	1.707 to 5.887	<0.001
Inotropic drugs prior to cardiac arrest	2.191	1.194 to 4.020	0.011
Sodium bicarbonate administration during resuscitation	3.241	1.850 to 5.677	<0.001
PaCO_2_ < 30 mmHg 1 h after ROSC	2.623	1.076 to 6.397	0.034
PaCO_2_ > 50 mmHg 1 h after ROSC	2.004	1.011 to 3.970	0.046
FiO_2_ ≥ 0.80 24 hours after ROSC	4.611	1.934 to 10.993	<0.001

## Discussion

To our knowledge, this is the first multicenter multinational study that analyzed the association of early post-ROSC factors with outcome of in-hospital cardiac arrest in children according to the Utstein style guidelines. Sustained ROSC was achieved in 69.5% patients but secondary in-hospital mortality among the initial survivors of CA was 43.5% and survival to hospital discharge was therefore 39.2%.

### Oxygenation and ventilation parameters

Several studies, including our previous analysis, showed that alterations in ventilation and oxygenation during the first hours after ROSC are associated with prognosis [14,20-24]. Our study shows that PaCO_2_ < 30 mmHg and >50 mmHg at 1 hour and PaCO_2_ > 50 mmHg at 24 hours after ROSC are mortality indicators [14]. Our results differ from those reported in a retrospective study in 195 children after CA, in which no relationship was found between ventilation and mortality [20]. Hyperventilation may increase mortality and brain damage by reducing cerebral blood flow and tissue perfusion resulting in ischemia [22]. On the other hand, hypoventilation may increase the risk of cerebral edema and intracranial hypertension due to cerebral vasodilation [23]. In addition, hypercapnia can impair myocardial function and induce vasoconstriction of the pulmonary vascular bed [23]. Our findings highlight the importance of monitoring ventilation using capnography and blood gas analysis in order to rapidly achieve an appropriate ventilation status after ROSC, although capnography values can be altered in patients with abnormal PaO_2_/FiO_2_.

PaO_2_ was not associated with mortality in the univariate or in the multivariate analysis at 1 hour and at 24 hours after ROSC. Two recent retrospective studies in children did not find this association between mortality and oxygenation either [20,24]. Nevertheless, another retrospective study that analyzed 1,875 pediatric patients found a correlation between mortality in the PICU and hypoxia and, to a lesser extent, with hyperoxia. This study did not analyze the relationship between ventilation and mortality [21].

In our study, non-survivors had higher FiO_2_ than survivors, and the univariate analysis showed that FiO_2_ ≥ 0.80 was associated with mortality. The multivariate logistic regression study showed that high FiO_2_ could be considered a risk factor only at 24 hours after ROSC. Elevated FiO_2_ may cause cellular toxicity as shown in previous studies in neonates [25]. On the other hand, elevated FiO_2_ could also indicate a greater need for oxygen, as worse tissue oxygenation may exist. Nevertheless, no relationship was found between PaO_2_ or PaO_2_/FiO_2_ and mortality in the patients in our study, and it may be that these patients did not require such a high FiO_2_. On the other hand we did not find association between ventilation and oxygenation parameters and neurologic outcome. This may be because the number of patients with hypoxia and hyperoxia was insufficient to detect significant differences, or that only important alterations in oxygenation could influence neurologic outcome.

We think that it is possible that ventilation and oxygenation could influence the prognosis of children who suffer CA. However, multicenter controlled studies with a sufficient number of patients are needed because many other factors besides ventilation and oxygenation may influence outcome in CA patients.

### Lactid acid

Lactic acid is one of the most commonly used parameters to assess and monitor hypoperfusion or tissue hypoxia in critically ill patients, as it has been demonstrated to have good prognostic capacity and it is easy and fast to measure [26]. Lactate levels in patients who have recovered from CA probably reflect the severity of the ischemia-reperfusion syndrome. Nevertheless, high lactic acid levels may exist without the presence of tissue hypoperfusion due to the administration of adrenaline or to the presence of hyperglycemia, which are very common after CA [26].

Several studies have found that lactate levels in the first 48 hours after CA is lower in survivors and in patients without neurological damage [27-29]. The levels of lactate after ROSC and 12 or 24 h later were significantly higher in non survivors adults and children after out-of-hospital and in-hospital CA [9,30-33]. Lactate clearance within the first 24 hours (lactate after ROSC minus lactate 24 hours after ROSC) × 100/lactate after ROSC) is significantly higher in survivors than in non-survivors [30-32].

In our study, non-survivors had more acidosis both at 1 hour and at 24 hours after ROSC. Acidosis was mainly due to metabolic acidosis, with lower bicarbonate levels and higher base deficit in non-survivors than in survivors. Nevertheless, the only factor associated with mortality in the logistic regression analysis was lactic acid at 1 hour and at 24 hours after ROSC. Non-survivors presented significantly higher levels of lactic acid at 1 and 24 hours after ROSC, and lactic acid levels >5 mmol/L were associated with higher mortality in the univariate and multivariate analysis.

Although lactate at 1 and at 24 hours after ROSC was higher in non-survivors, no significant differences were found in lactate clearance, because lactate acid levels significantly decreased in the first 24 hours in both groups (from 16.9 to 5.8 mmol/L in non-survivors and from 6.7 to 3.7 mmol/L in survivors).

### Vasoactive treatment

Hemodynamic alterations after ROSC are also late mortality risk factors. Cardiac rhythm after ROSC and need for vasoactive drugs in the first 24 hours were analyzed in order to assess hemodynamic alterations. Other hemodynamic parameters, such as heart rate, blood pressure or central venous pressure were not registered. The non-surviving group had a greater percentage of patients requiring vasoactive support and at higher doses (higher inotropic index) than the surviving group. The univariate and multivariate studies showed that vasoactive-inotropic score >14 was significantly associated with mortality.

Several studies have found that the need for pressors previous to CA is a mortality risk factor, both in adults [34-37] and in children [4,6,8,11]. Our study shows that the need for pressors and at higher doses (vasoactive-inotropic score) after CA is associated with higher risk of mortality. This fact has also been found by Meert *et al*. [9]. This highlights the influence of early hemodynamic alterations on outcome in children after CA, and the importance of treating these alterations as they appear.

On the other hand, a large percentage of patients received more than one vasoactive agent after ROSC. That may be the cause for not finding a significant association between mortality and the administration or dosage of any specific vasoactive drug, but with the intensity of vasoactive treatment in general. Inotropic score has proved to be appropriate in assessing vasoactive support and its relationship with mortality in several studies in children in shock, after open heart surgery and after heart transplantation [18,38,39]. Rhodes *et al.* found that the inotropic score was higher in non-survivors than in survivors with CA after congenital heart surgery [39]. Our study also suggests that the vasoactive-inotropic score may be a useful prognostic indicator in children after CA.

A recent retrospective study in adults showed that the combination of elevated lactate levels and the need for vasoactive support had a good mortality predictive capacity in patients that recovered from CA [40]. Our results agree with those from the mentioned study, although in our study the dose of vasoactive support also proved to have mortality predictive capacity. Furthermore, PaCO_2_ levels, which were not registered in the study in adults, also proved to have prognostic capacity in our study. On the other hand, lactic acid levels >5 mmol/L at 1 hour after ROSC was the only factor that was associated with bad neurological outcome in our study.

### Pre-arrest, resuscitation and post-ROSC multivariate analysis

Meert *et al*. [9] performed a multivariate analysis including pre-arrest, resuscitation and 12 h post-ROSC factors. In this study the only post-ROSC factor associated with survival al hospital discharge was the responsive pupils after ROSC. Conversely, our multivariate analysis, including pre-arrest, resuscitation and post-ROSC factors, showed that the post-ROSC factors associated with mortality were hypoventilation and hyperventilation 1 hour after ROSC and high FiO_2_ 24 hours after ROSC, highlighting the importance of the control of ventilation and oxygenation after ROSC.

### Limitations

Our study has several limitations. One of them is that hemodynamic variables such as heart rate, blood pressure and central venous pressure in the first 24 hours after ROSC were not registered, making it impossible to accurately assess the presence of shock in these patients. In a recent study hypotension after ROSC was related to bad prognosis [41]. Hypothermia or hyperthermia and the parameters of mechanical ventilation were not registered neither.

Pre-arrest values of lactate or vasoactive-inotropic score could influence post-ROSC values but we did not register pre-arrest lactate and VIS data in our patients. On the other hand, our study has only analyzed prognostic factors in the first 24 hours after ROSC, which may be the most important but not the only ones. Other factors that affect prognosis but may appear in the following days, such as nosocomial infections or multiple organ failure, were not analyzed. Actually, the median PICU stay for non-survivors was 8 days, and many of these patients died because of complications due to multi-organ failure.

Finally, only 61% of patients have neurologic outcome evaluation, although there were no differences in baseline characteristics between patients with and without a neurologic outcome measure. On the other hand, only a small number of patients had bad neurological outcomes. This is why the power of the statistical analysis in relationship with neurological outcome is poor, so results must be interpreted with caution. Therefore, more studies are needed to prospectively assess both early and late post-ROSC mortality and neurologic outcome risk factors in children after CA.

## Conclusions

We conclude that secondary in-hospital mortality among the initial survivors of CA is high (43.5% in our study). The most important early mortality risk factors after ROSC in in-hospital CA in children are hyperventilation, hypoventilation, high FiO_2_ requirements need for high doses of inotropic drugs and high lactic acid levels. High lactic acid levels at 1 hour after ROSC were associated with bad neurological outcome.

## Key messages

- Secondary in-hospital mortality among the initial survivors of CA is high (43.5% in our study)
- The most important early mortality risk factors after ROSC in-hospital CA in children were hyperventilation, hypoventilation, need for high doses of inotropic drugs, high lactic acid levels and high FiO_2_ requirements
- High lactic acid levels at 1 hour after ROSC were associated with bad neurological outcome
- Early treatment of hemodynamic and respiratory disturbances after ROSC could improve mortality in initial survivors of CA

## Additional file

Additional file 1:
**List of Hospital Review Boards.**

## Abbreviations

BE: base excess
bpm: beats per minute
CA: cardiac arrest
CPR: cardiopulmonary resuscitation
ECG: electrocardiographic
ECMO: extracorporeal membrane oxygenation
FiO_2_: inspired oxygen fraction
OR: adjusted odds ratios
PaO_2_: arterial partial pressure of oxygen
PCPC: pediatric cerebral performance category
PICU: Pediatric Intensive Care Unit
ROC: receiver operator characteristic
ROSC: return of spontaneous circulation
VIS: vasoactive-inotropic index
