# Supplementary material for: Post return of spontaneous circulation factors associated with mortality in pediatric in-hospital cardiac arrest: a prospective multicenter multinational observational study
Source: Crit Care. 2014 Nov 3;18(6):607. doi: 10.1186/s13054-014-0607-9 (PMC4245792; doi:10.1186/s13054-014-0607-9)
Supplement: Additional file 1: — List of Hospital Review Boards. [file 13054_2014_607_MOESM1_ESM.doc]

Additional file 1

**List of Hospitals Review Boards**

Hospital General Universitario Gregorio Maranon, Madrid, Spain; Hospital Escuela, Tegucigalpa, Honduras; Hospital Valle de Hebron, Barcelona, Spain; Hospital Nino Jesus, Tucuman, Argentina;Ospedale Bambinu Gesu, Roma, Italy; Hospital San Joao, Porto, Portugal; Irmandade da Santa Casa de Misericordia, Sao Paulo, Brasil; Hospital de Cruces, Baracaldo, Spain; Hospital de Ninos Ricardo Gutierrez, Buenos Aires, Argentina; Hospital Clinico Universitario, Santiago de Compostela, Spain; Hospital Pediatrico, Coimbra, Portugal; Hospital Nino Jesus, Madrid, Spain; Ospedale Gaslini, Genova, Italy; Hospital Universitario La Paz, Madrid, Spain; Hospital Pablo Tobon Uribe, Medellin, Colombia; Hospital Britanico, Buenos Aires, Argentina; Hospital Reina Sofia, Cordoba, Spain; Hospital Virgen de la Salud, Toledo, Spain; Hospital Roberto del Rio, Santiago de Chile, Chile; Hospital de Cabueñes, Asturias, Spain; Hospital Universitario Austral, Buenos Aires, Argentina; Hospital Nacional de Asunción, Asuncion, Paraguay; Hospital Son Dureta, Palma de Mallorca, Spain; Hospital Central de Asturias, Oviedo, Spain; Hospital Carlos Haya, Málaga, Spain; Hospital Materno Infantil de Las Palmas, Las Palmas de Gran Canaria, Spain; Hospital da Clinicas da UFPR, Curitiba, Brasil; Hospital SOLCA, Quito, Ecuador; Complexo Hospitalario Universitario de Vigo, Vigo, Spain; Corporacion Parc Taul, Sabadell, Spain; Hospital Garcia de Orta, Almada, Portugal; Hospital Regional Rio Gallegos, Rio Gallegos, Argentina; Hospital San Juan de Dios, Barcelona, Spain; Complejo Asistencial de Burgos, Spain; Hospital Son Dureta, Palma de Mallorca, Spain; Hospital Josep Trueta, Gerona, Spain; Hospital de Cabueñes, Asturias, Spain; Hospital Virgen del Camino, Pamplona, Spain; Hospital Clínico de Valladolid, Spain; Hospital Mutua de Tarrasa, Barcelona, Spain; Hospital Universitario de Canarias, Tenerife, Spain; Hospital Virgen del Rocío, Sevilla, Spain; Hospital Germans Trias i Pujol, Barcelona, Spain; Hospital Oscar Alende, Lomas de Zamora, Vicente López y Planes, General Rodriguez, Argentina; Hospital de Clínicas UBA, Argentina; Clínica Shaio, Bogota, Colombia; Hospital Infantil de México Federico Gómez, Mexico, Spain.
